# In Vivo and Computational Studies on Sitagliptin’s Neuroprotective Role in Type 2 Diabetes Mellitus: Implications for Alzheimer’s Disease

**DOI:** 10.3390/brainsci14121191

**Published:** 2024-11-26

**Authors:** Vasudevan Mani, Minhajul Arfeen

**Affiliations:** 1 Department of Pharmacology and Toxicology, College of Pharmacy, Qassim University, Buraydah 51452, Saudi Arabia; 2 Department of Medicinal Chemistry and Pharmacognosy, College of Pharmacy, Qassim University, Buraydah 51452, Saudi Arabia; m.arfeen@qu.edu.sa

**Keywords:** sitagliptin, type 2 diabetes mellitus, Alzheimer’s disease, brain enzymes, inflammation, oxidative stress, apoptosis, molecular docking

## Abstract

Background/Objectives: Diabetes mellitus (DM), a widespread endocrine disorder characterized by chronic hyperglycemia, can cause nerve damage and increase the risk of neurodegenerative diseases such as Alzheimer’s disease (AD). Effective blood glucose management is essential, and sitagliptin (SITG), a dipeptidyl peptidase-4 (*DPP-4*) inhibitor, may offer neuroprotective benefits in type 2 diabetes mellitus (T2DM). Methods: T2DM was induced in rats using nicotinamide (NICO) and streptozotocin (STZ), and biomarkers of AD and DM-linked enzymes, inflammation, oxidative stress, and apoptosis were evaluated in the brain. Computational studies supported the in vivo findings. Results: SITG significantly reduced the brain enzyme levels of acetylcholinesterase (*AChE*), beta-secretase-1 (*BACE-1*), *DPP-4*, and glycogen synthase kinase-3β (*GSK-3β*) in T2DM-induced rats. It also reduced inflammation by lowering cyclooxygenase-2 (*COX-2*), prostaglandin E2 (PGE2), tumor necrosis factor-α (TNF-α), and nuclear factor-κB (NF-κB). Additionally, SITG improved oxidative stress markers by reducing malondialdehyde (MDA) and enhancing glutathione (GSH). It increased anti-apoptotic B-cell lymphoma protein-2 (Bcl-2) while reducing pro-apoptotic markers such as Bcl-2-associated X (BAX) and Caspace-3. SITG also lowered blood glucose levels and improved plasma insulin levels. To explore potential molecular level mechanisms, docking was performed on *AChE*, *COX-2*, *GSK-3β*, *BACE-1*, and Caspace-3. The potential binding affinity of SITG for the above-mentioned target enzymes were 10.8, 8.0, 9.7, 7.7, and 7.9 kcal/mol, respectively, comparable to co-crystallized ligands. Further binding mode analysis of the lowest energy conformation revealed interactions with the critical residues. Conclusions: These findings highlight SITG’s neuroprotective molecular targets in T2DM-associated neurodegeneration and its potential as a therapeutic approach for AD, warranting further clinical investigations.

## 1. Introduction

Diabetes is a complex metabolic disorder characterized by elevated blood glucose levels due to defects in insulin secretion or the body’s response to insulin. It is primarily classified into two types: type 1 diabetes, which requires insulin administration and accounts for 5–10% of cases; and type 2 diabetes, an insulin-independent form affecting up to 90% of individuals with diabetes [1]. The global prevalence of diabetes has increased dramatically, rising from 366 million cases in 2011 to over 536.6 million in 2021, with projections estimating 783.2 million cases by 2045 [2]. Saudi Arabia ranks sixth globally in diabetes prevalence, with 23.9% of its population affected [3]. This escalating prevalence carries significant public health and economic consequences.

Moreover, DM is associated with various complications, including cardiovascular disease, retinal damage, nephropathy, and neurotoxicity. Epidemiological studies indicate that diabetic patients have a heightened risk of neurotoxicity, memory impairment, and increased susceptibility to developing AD [4,5]. Disruptions in insulin and insulin-like growth factor signaling contribute to neurodegeneration by activating pathways that promote inflammation and cell death [6]. Among these, apoptosis is a key mechanism by which hyperglycemia induces neuronal death, possibly due to altered gene expressions regulating cell survival. The Bcl-2 protein family plays a crucial role in apoptosis regulation, where Bcl-2 supports cell survival and Bax promotes cell death. Caspace-3, a major mediator of neuronal apoptosis, is also involved in diabetes and various neurodegenerative diseases, including AD [7,8]. Additionally, *GSK-3β*, the predominant isoform from the GSK-3 family, plays a critical role in both diabetes and neurodegenerative processes [9]. In DM, *GSK-3β* is a key factor contributing to insulin deficiency and insulin resistance. It is also widely expressed throughout the brain, where it promotes tau hyperphosphorylation and amyloid-β (Aβ) production through β-secretase (*BACE-1*) and γ-secretase cleavage, leading to impaired learning and memory. Moreover, *GSK-3β* may exacerbate microglial-driven inflammatory responses around Aβ plaques [9,10].

Interestingly, T2DM and AD share notable pathological similarities, such as Aβ deposition, insulin resistance, oxidative stress, and chronic inflammation [4]. Emerging research highlights the overlap between these conditions, suggesting that treatments for T2DM, particularly *DPP-4* inhibitors, may have potential in addressing AD pathology [11]. Sitagliptin (SITG), a widely prescribed *DPP-4* inhibitor for T2DM, enhances postprandial insulin release by preventing the degradation of incretin hormones. This action improves insulin secretion, reduces hepatic glucose production, and lowers blood glucose levels [12]. Beyond its antidiabetic properties, SITG has demonstrated neuroprotective effects in T2DM models, although these have been less extensively documented. Recent studies have indicated SITG’s capacity to slow AD progression by reducing amyloid deposits in transgenic AD mouse models [13]. Additionally, when combined with quercetin, SITG enhanced cognitive function in AD rat models by reducing Aβ1–42 levels, increasing antioxidant activity and upregulating the Nrf2/HO-1 pathway [14]. Moreover, SITG also enhanced memory performance in rats with Parkinson’s disease (PD) by increasing levels of BDNF, which helps to protect against neuronal loss and the deterioration of dendritic spines [15]. Treatment with both SITG and liraglutide prevented the progression of PD by protecting nigral neuronal pathways from inflammatory and apoptotic damage [16]. These findings are further supported by SITG’s positive effects on memory, including improved working and reference memory, reductions in glucose and insulin levels, and increased acetylcholine (ACh) content in the hypothalamus [17]. SITG has also been shown to mitigate tau aggregation, promote Aβ degradation, and reverse neurodegenerative processes similar to AD. These effects are partly mediated by the restoration of the glucagon-like peptide-1 (GLP-1) signaling pathway, particularly through the MAPK and PI3K-Akt pathways [18].

These observations underscore SITG ‘s potential as a therapeutic option for targeting AD-related pathological changes in diabetic conditions, particularly with early intervention. This connection highlights the intricate relationship between T2DM and AD, suggesting new therapeutic possibilities. Therefore, this study aims to explore the protective mechanisms of SITG against neurotoxicity induced by type 2 diabetes in rat models.

## 2. Materials and Methods

### 2.1. Animals

For this study, 30 adult male Sprague Dawley rats, aged 11 to 12 weeks and weighing 150–170 g, were sourced from the animal facility at the College of Pharmacy, Qassim University, KSA. The rats were divided into five groups, with six animals in each group. Ethical approval for animal use was granted by the Health Research Ethics Committee under the Deanship of Scientific Research at Qassim University (approval number 23-60-16; grant number 2023-SDG-1-HMSRC-35884). The rats were accommodated in groups of three per cage, maintained on a 12-h light/dark cycle, with free access to food and water. A one-week acclimatization period was observed before starting the drug treatment.

### 2.2. Chemicals

Sitagliptin phosphate monohydrate (SITG, Cat. No.: A323507), streptozotocin (STZ, Cat. No.: A667625), and nicotinamide hydrochloride (NICO; Cat. No.: A1671233) were purchased from Ambeed, Arlington Heights, IL, USA. All ELISA kits used in the experiments were obtained from MyBioSource, San Diego, CA, USA. SITG and NICO were dissolved in normal saline (NS), while STZ was dissolved in a cold, freshly prepared citrate buffer (0.1 M; pH 4.5) immediately before administration.

### 2.3. Type 2 Diabetes Mellitus (T2DM) Induction

The rats underwent a 12-h fasting period prior to the injection procedure. T2DM was induced by an injection of NICO (120 mg/kg, i.p.), followed 15 min later by an STZ injection (60 mg/kg, i.p.). This sequential protocol facilitates the onset of diabetes. Hyperglycemia was assessed to confirm diabetes induction, with blood glucose levels measured 72 h post-injection. Rats with fasting blood glucose levels above 126 mg/dL were considered diabetic [19,20]. Blood glucose levels were assessed using an Accu-Chek glucometer (Roche, Germany).

### 2.4. Experimental Groups

**Figure 1:**
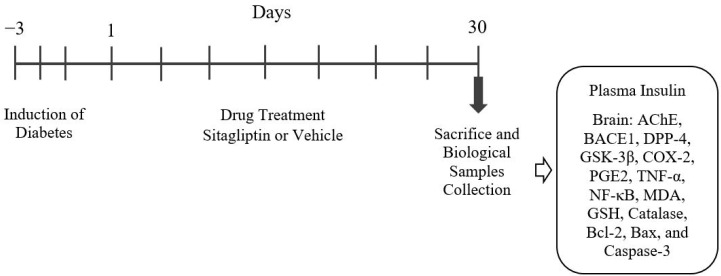
The timeline of the drug treatment and the experiment schedule.

The rats were assigned to five groups for the experiment: Group 1 (Normal control) received the vehicle orally for 30 days; Group 2 (SITG 30) was administered SITG at a dose of 30 mg/kg orally for 30 days; Group 3 (Diabetic) had T2DM induced using STZ (60 mg/kg, i.p.) and NICO (120 mg/kg, i.p.), followed by vehicle administration for 30 days (p.o.); Group 4 (Diabetic + SITG10) was treated with SITG (10 mg/kg, p.o.) for 30 days following T2DM induction; Group 5 (Diabetic + SITG 30) also had T2DM induced and was administered SITG (30 mg/kg, p.o.) for 30 days (Figure 1). The SITG [15,16], STZ, and NICO [19,20] doses were determined from previous research findings. During the treatments, the bodyweight of the rats and blood glucose levels were measured on days 1, 15, and 30.

### 2.5. Blood and Brain Samples Collection

On the 30th day, at the end of the treatment cycle, blood and brain tissues were collected from each animal for targeted protein quantification (Figure 1). Prior to collection, each rat was euthanized via cervical decapitation under mild anesthesia [ketamine (100 mg/kg, i.p.) and xylazine (10 mg/kg, i.p.)]. EDTA-coated tubes were used to collect the blood samples and plasma was separated by centrifugation at 4000 rpm for 10 min to quantify insulin levels. Following blood collection, the brain was carefully extracted from each rat. The whole brain was homogenized in phosphate-buffered saline (ice-cold, pH 7.4), followed by centrifugation to collect the supernatant. The supernatant underwent total protein quantification using the biuret colorimetric assay and was further analyzed for various biochemical parameters as detailed below.

### 2.6. Quantification of Plasma Insulin

Plasma insulin levels were assessed using a rat insulin ELISA kit that employs the competitive inhibition enzyme immunoassay technique (Catalog No.: MBS2700141). Briefly, 50 µL of plasma was mixed with 150 µL of the working reagent in individual wells of a 96-well plate pre-coated with a specific insulin monoclonal antibody. Following the standard protocol, absorbance readings were taken at 450 nm.

### 2.7. Quantification of Targeted Enzymes

Brain homogenates were analyzed for neurodegeneration-related enzyme levels, including *AChE* (Catalog No.: MBS2709297), BACE1 (Catalog No.: MBS2886958), *GSK-3β* (Catalog No.: MBS284677), and *DPP-4* (Catalog No.: MBS2020584), using targeted ELISA assay kits. The assays followed the double-antibody sandwich method. Briefly, 100 µL of each sample was added to wells pre-coated with specific antibodies, and standard procedures from the product catalog were followed. The optical density of the color change was measured at 450 nm.

### 2.8. Quantification of Neuroinflammation Parameters

To analyze neuroinflammatory parameters, specific ELISA kits were used to measure the levels of the *COX-2* enzyme, prostaglandin PGE2, pro-inflammatory cytokine TNF-α, and the transcription factor NF-κB. The assays followed the following principles: *COX-2* (Catalog No.: MBS725633) was assessed using a competitive enzyme immunoassay technique with a polyclonal anti-*COX-2* antibody; PGE2 (Catalog No.: MBS267737) employed a competitive ELISA detection method with antigen-coated wells; TNF-α (Catalog No.: MBS267737) followed a double-sandwich ELISA technique pre-coated with a rat TNF-α monoclonal antibody; NF-κB (Catalog No.: MBS453975) was analyzed using a quantitative sandwich ELISA pre-coated with an antibody specific to NF-κB. Each assay was conducted as per the procedures outlined in the product catalogs.

### 2.9. Quantification of Oxidative Markers

The oxidative marker MDA (Catalog No.: MBS268427) and two antioxidant markers, GSH (Catalog No.: MBS265966) and catalase (Catalog No.: MBS2704433), were quantified from brain homogenates using specific rat ELISA kits. All three assays employed the double-antibody sandwich ELISA technique, with 96-well plates pre-coated with anti-rat MDA, GSH, or catalase monoclonal antibodies. The standard assay procedures, as outlined in the respective catalogs, were followed for sample analysis.

### 2.10. Quantification of Apoptosis Proteins

The levels of apoptotic regulators, including the anti-apoptotic protein Bcl-2 (Catalog No.: MBS452319) as well as pro-apoptotic proteins Bax (Catalog No.: MBS165136) and Caspace-3 (Catalog No.: MBS261814), were determined using specific rat ELISA kits. The assays followed a quantitative sandwich ELISA method, with each kit pre-coated with antibodies specific to Bcl-2, Bax, or Caspace-3. The standard procedures were followed as per the corresponding kit catalogs.

### 2.11. Molecular Docking

Molecular docking studies were performed using AutoDock Vina (AutoDock Vina, CA, USA), as in our previous studies. Preparation of the input files was managed through AutoDock Tools, which are included with MGL Tools (version 1.5.6, La Jolla, CA, USA). The three-dimensional structure of SITG was obtained from the ZINC15 database in SDF format [21,22].

### 2.12. Statistical Analysis

Statistical analysis was performed using GraphPad Prism version 9.5.0 (GraphPad Software Inc., San Diego, CA, USA). The data were expressed as mean values along with the standard error of the mean (SEM) to represent variability. A one-way ANOVA was conducted to assess differences among the groups, followed by the Tukey–Kramer post hoc test to identify specific pairwise comparisons. Statistical significance was determined with a threshold of *p* ≤ 0.05 for all tests.

## 3. Results

### 3.1. Induction of Diabetes and Effect of SITG Treatment on Rat’s Body Weight

**Figure 2:**
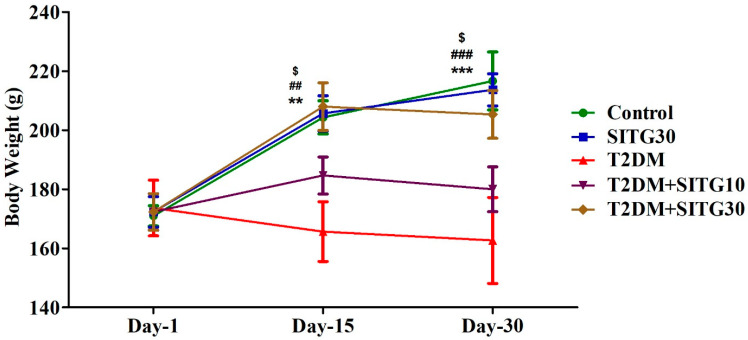
Effect of diabetes and sitagliptin on body weight in rats over a 30-day treatment period (*n* = 6). Data are presented as mean ± SEM. ** *p* < 0.01 and *** *p* < 0.001 vs. Day-1 in Control; ## *p* < 0.01 and ### *p* < 0.001 vs. Day 1 in SITG10; $ *p* < 0.05 vs. Day 1 in T2DM + SITG30.

To assess the effects of T2DM induction and SITG administration on body weight and the maintenance of other physiological parameters, animals were initially selected with no significant differences between groups before diabetes induction and on Day 1 post-induction. Throughout the treatment period (Figure 1), a gradual increase in body weight was observed in the control group as the days progressed, with significant changes noted on Day 15 (*p* < 0.01) and Day 30 (*p* < 0.001) compared to Day 1 (Figure 2). A similar pattern was observed in the SITG30 group, where body weight also significantly increased on Day 15 (*p* < 0.01) and Day 30 (*p* < 0.001) relative to Day 1. The T2DM + SITG30 group also showed a significant increase in body weight (*p* < 0.05) on both Day 15 and Day 30 compared to Day 1. However, the T2DM and T2DM + SITG10 groups exhibited no significant changes in body weight relative to their respective Day-1 values.

### 3.2. SITG Treatment Reduced the Blood Glucose Levels in T2DM-Induced Rats

**Figure 3:**
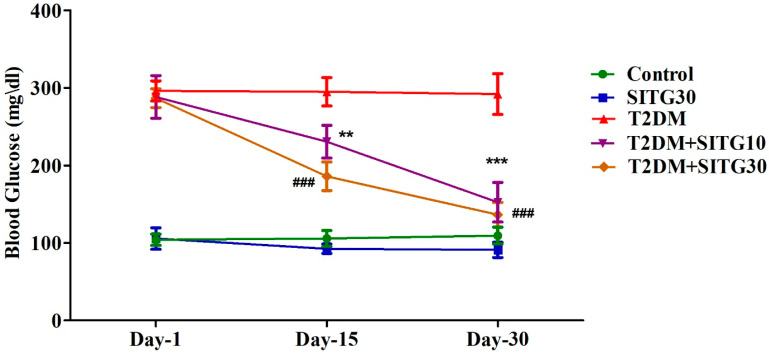
Effect of sitagliptin on blood glucose levels in diabetes-induced rats (*n* = 6). Data are presented as mean ± SEM. ** *p* < 0.01 and *** *p* < 0.001 vs. Day 1 in T2DM + SITG10; ### *p* < 0.001 vs. Day 1 in T2DM + SITG30.

The effects of SITG on blood glucose levels in normal and diabetes-induced rats are shown in Figure 3. To ensure consistency in parameter results, blood glucose levels were similar across all three T2DM-induced groups on Day 1. During the 30-day treatment cycle (Figure 1), the control, SITG30, and T2DM groups showed no significant changes in blood glucose levels on Day 15 and Day 30 compared to their corresponding Day-1 levels. However, SITG treatment significantly reduced blood glucose levels in T2DM-induced rats throughout the treatment period. Specifically, in the T2DM + SITG10 group, blood glucose levels were reduced with significance on Day 15 (*p* < 0.01) and Day 30 (*p* < 0.001) compared to Day-1 values. Similarly, the T2DM + SITG30 group exhibited an extensive reduction in blood glucose levels (*p* < 0.001) on both Day 15 and Day 30 relative to Day 1.

### 3.3. SITG Treatment Increased Plasma Insulin Levels in T2DM-Induced Rats

**Figure 4:**
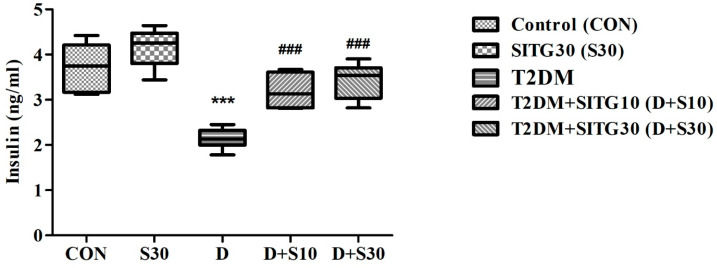
Effect of sitagliptin on plasma insulin levels in diabetes-induced rats (*n* = 6). Data are presented as mean ± SEM. *** *p* < 0.001 vs. Control; ### *p* < 0.001 vs. T2DM.

Measurement of plasma insulin levels is a critical marker in T2DM experiments, offering key insights into insulin dynamics, disease progression, and treatment effects. As shown in Figure 4, one-way ANOVA analysis revealed significant differences in plasma insulin levels (ng/mL) between the groups (F(4,25) = 21.70, *p* < 0.001) due to T2DM induction and SITG treatments. Specifically, diabetes induction (T2DM) significantly reduced plasma insulin levels to 2.140 ± 0.091 (*p* < 0.001) from the control value of 3.725 ± 0.208. However, treatments with T2DM + SITG10 and T2DM + SITG30 for 30 days significantly improved insulin levels to 3.192 ± 0.151 (*p* < 0.001) and 3.423 ± 0.161 (*p* < 0.001), respectively, compared to the diabetes-induced group. In SITG30-treated animals, plasma insulin levels increased to 4.153 ± 0.174, which exceeded the control value but did not reach statistical significance compared to the control group.

### 3.4. SITG Treatment Reduced Neurodegenerative Enzyme Levels in the Brains of T2DM-Induced Rats

**Figure 5:**
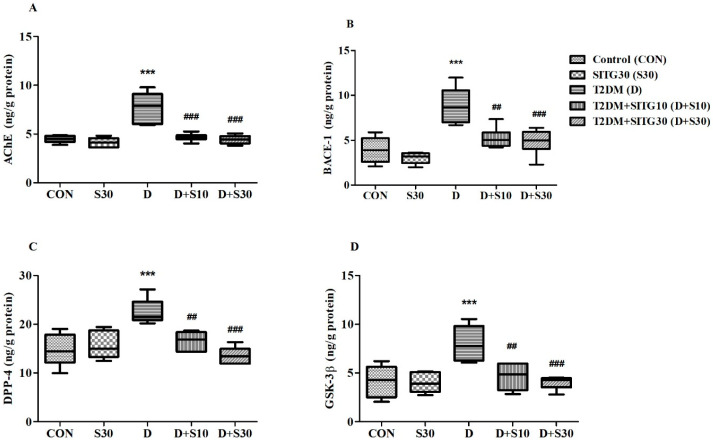
Effect of sitagliptin on enzyme activity in the brains of diabetes-induced rats (*n* = 6): (**A**) *AChE*, (**B**) *BACE-1*, (**C**) *DPP-4*, and (**D**) *GSK-3β*. Data are presented as mean ± SEM. *** *p* < 0.001 vs. Control; ## *p* < 0.01 and ### *p* < 0.001 vs. T2DM.

Figure 5 illustrates the effect of T2DM induction followed by SITG treatment on key enzymes in the rat brain: *AChE*, *BACE-1*, *DPP-4*, and *GSK-3β*. These enzymes are linked to diabetes-induced neurodegeneration by driving processes such as cholinergic deficiency (*AChE*), amyloid plaque formation (*BACE-1*), inflammation and oxidative stress (*DPP-4*), and tau pathology (*GSK-3β*). The figure highlights how SITG treatment mitigates these enzyme activities, potentially reducing diabetes-related neurodegenerative damage.

In Figure 5A, a comparison between the treatment groups reveals significant alterations in brain *AChE* levels (ng/g protein) following T2DM induction and subsequent SITG treatment (F(4,25) = 21.38, *p* < 0.001). T2DM induction led to a marked increase in *AChE* levels (7.751 ± 0.629; *p* < 0.001) compared to control animals (4.485 ± 0.147). After SITG treatment, *AChE* levels were significantly reduced in the T2DM + SITG10 (4.671 ± 0.160; *p* < 0.001) and T2DM + SITG30 (4.433 ± 0.183; *p* < 0.001) groups in diabetes rats, restoring them to levels comparable to control animals. Additionally, *AChE* levels in the SITG30 group (4.151 ± 0.214) closely matched those of the control group.

In terms of *BACE-1* enzyme levels (ng/g protein) in the rat brain, analysis (F(4,25) = 15.38, *p* < 0.001) indicates significant modulation by the treatments (Figure 5B). T2DM induction caused a notable increase in *BACE-1* levels, reaching 8.857 ± 0.801 (*p* < 0.001) compared to the control group (3.917 ± 0.584). However, treatment with SITG significantly reduced *BACE-1* levels, with T2DM + SITG10 bringing them down to 5.238 ± 0.471 (*p* < 0.01) and T2DM + SITG30 lowering them further to 4.835 ± 0.583 (*p* < 0.001), relative to the T2DM group. The SITG30 group recorded *BACE-1* levels at 3.022 ± 0.268, which were comparable to the control group.

The analysis of *DPP-4* levels (ng/g protein) among the experimental groups revealed significant differences (F(4,25) = 11.71, *p* < 0.001) due to the treatments (Figure 5C). In control animals, *DPP-4* levels were 14.71 ± 1.338, but T2DM induction caused a notable increase to 22.55 ± 1.053. After treatment, these elevated levels decreased to 16.57 ± 0.769 (*p* < 0.01) in the T2DM + SITG10 group and further declined to 13.58 ± 0.699 (*p* < 0.001) in the T2DM + SITG30 group. The SITG30 group recorded *DPP-4* levels of 15.65 ± 1.122.

Similar to the other enzymes, *GSK-3β* levels (ng/g protein) were significantly influenced (F(4,25) = 9.621, *p* < 0.001) across the treatment groups (Figure 5D). In T2DM animals, *GSK-3β* levels were elevated to 8.014 ± 0.729, which was significantly higher (*p* < 0.001) compared to control animals (4.153 ± 0.660). Following treatment, *GSK-3β* levels were effectively reduced in both treatments, T2DM + SITG10 (4.652 ± 0.575, *p* < 0.01) and T2DM + SITG30 (4.012 ± 0.269, *p* < 0.001), when matched to the T2DM group. The SITG30 group exhibited *GSK-3β* levels (3.999 ± 0.418) similar to those in control animals.

### 3.5. SITG Treatment Reduced Neuronal Inflammation in the Brains of T2DM-Induced Rats

**Figure 6:**
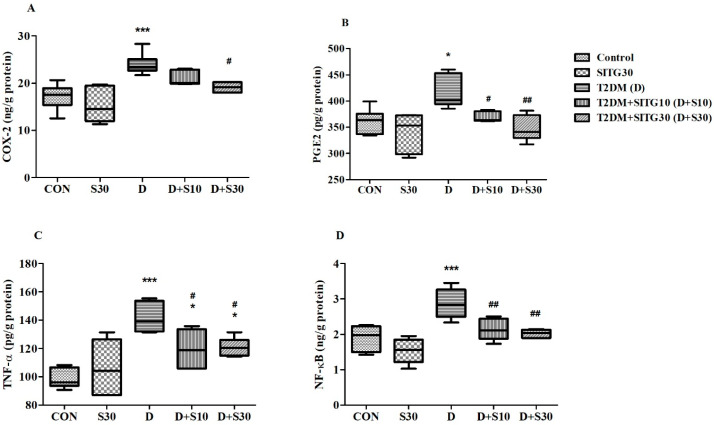
Effect of sitagliptin on inflammatory markers in the brains of diabetes-induced rats (*n* = 6): (**A**) *COX-2*, (**B**) PGE2, (**C**) TNF-α, and (**D**) NF-κB. Data are presented as mean ± SEM. * *p* < 0.05 and *** *p* < 0.001 vs. Control; # *p* < 0.05 and ## *p* < 0.01 vs. T2DM.

To highlight the impact of SITG on neuroinflammation induced by diabetes, four key inflammatory markers were studied: *COX-2*, PGE-2, TNF-α, and NF-κB (Figure 6). These targets are recognized as crucial players in the process of neuronal inflammation and are instrumental in understanding the inflammatory response associated with neurodegeneration in diabetic conditions.

In neuroinflammation, the *COX-2* enzyme plays a key role by catalyzing the production of pro-inflammatory prostaglandins, with elevated *COX-2* levels being directly linked to neuronal damage in the neurodegenerative process. The one-way ANOVA analysis revealed significant changes in *COX-2* levels (ng/g protein) across the treatment groups (F(4,25) = 11.03, *p* < 0.001) (Figure 6A). Specifically, T2DM-induced rats exhibited elevated *COX-2* levels (29.98 ± 0.928, *p* < 0.001), indicating neuronal damage, compared to control levels (17.15 ± 1.094). Among the treatment groups, the higher dose of SITG (T2DM + SITG30) effectively reduced *COX-2* levels (19.14 ± 0.435, *p* < 0.05) compared to T2DM rats. Further, the levels of *COX-2* were closer to the control group with SITG30 administration (15.29 ± 1.578).

In the inflammatory process, PGE2 promotes microglial activation and the release of pro-inflammatory cytokines, contributing to neuronal damage. Evidence from Figure 6B shows that PGE2 levels (pg/g protein) varied significantly between the experimental groups, as indicated by F(4,25) = 7.594 (*p* < 0.001). Specifically, compared to control rats (361.2 ± 9.658), T2DM rats exhibited elevated PGE2 levels (416.3 ± 12.77, *p* < 0.05). However, after 30 days of treatment, these elevated PGE2 levels were reduced in the T2DM + SITG10 (369.1 ± 3.934, *p* < 0.05) and T2DM + SITG30 (347.5 ± 9.847, *p* < 0.05) groups. The PGE2 levels in the SITG30 group were 340.8 ± 14.57, closer to the control group.

TNF-α is a pro-inflammatory cytokine that contributes to neuroinflammation by activating microglia and astrocytes, which subsequently leads to the release of further inflammatory mediators. One-way ANOVA analysis showed significant variations in TNF-α levels (pg/g protein) among the treatment groups, with F(4,25) = 11.29 (*p* < 0.001) (Figure 6C). In the control group, TNF-α levels were measured at 98.73 ± 2.825. In contrast, T2DM rats displayed a significant increase in TNF-α levels (141.8 ± 4.173, *p* < 0.001). Following treatment, the TNF-α levels were recorded at 119.7 ± 5.487 (*p* < 0.05) for the T2DM + SITG10 group and 121.0 ± 2.594 for the T2DM + SITG30 group. The SITG30 group exhibited TNF-α levels of 106.4 ± 7.593.

The transcription factor NF-κB regulates the expression of pro-inflammatory cytokines and mediators, playing a crucial role in the inflammatory processes associated with neurodegeneration. Consistent with the previously mentioned markers, NF-κB levels (ng/g protein) were significantly affected by the treatments, as indicated by F(4,25) = 11.29 (*p* < 0.001) (Figure 6D). The induction of diabetes was associated with elevated NF-κB levels (2.867 ± 0.168, *p* < 0.001) in the brains of T2DM rats compared to the control group (1.898 ± 0.144, *p* < 0.001). Furthermore, the effects of SITG were confirmed by a reduction in NF-κB levels in the T2DM + SITG10 (2.135 ± 0.127, *p* < 0.01) and T2DM + SITG30 (2.027 ± 0.047) groups compared to the T2DM group. In the SITG30 group, NF-κB levels were measured at 1.535 ± 0.149.

### 3.6. SITG Treatment Reduced Oxidative Stress in the Brains of T2DM-Induced Rats

**Figure 7:**
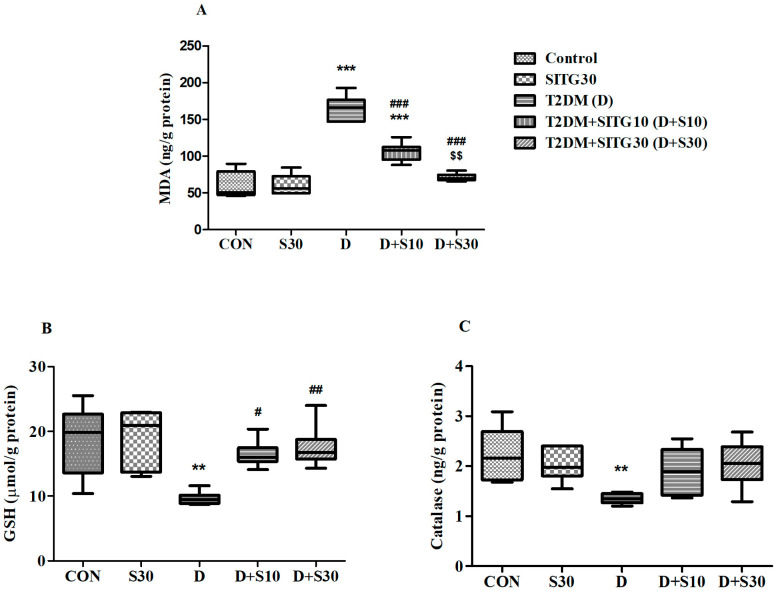
Effect of sitagliptin on oxidative and antioxidant markers in the brains of diabetes-induced rats (*n* = 6): (**A**) MDA, (**B**) GSH, and (**C**) Catalase. Data are presented as mean ± SEM. ** *p* < 0.01 and *** *p* < 0.001 vs. Control; # *p* < 0.05, ## *p* < 0.01 and ### *p* < 0.001 vs. T2DM; $$ *p* < 0.01 vs. T2DM + SITG10.

In this study, the oxidative stress marker MDA and two key antioxidant markers, GSH and catalase, were analyzed in brain samples to assess the potential of SITG in mitigating oxidative stress induced by diabetes in rats. The findings, as depicted in Figure 7, demonstrate the impact of SITG treatment on oxidative stress levels in the context of diabetes.

MDA, a byproduct of lipid peroxidation (LPO) resulting from ROS-induced cell membrane damage, serves as a key marker for oxidative damage. The overall analysis revealed significant changes in MDA levels (ng/g protein) between the treatment groups, as evidenced by F(4,25) = 16.53 (*p* < 0.001) (Figure 7A). A notable elevation in MDA levels (165.2 ± 6.983, *p* < 0.001) was observed in T2DM rats compared to the control group (60.95 ± 7.41), indicating diabetes-induced oxidative damage. However, both treatments, T2DM + SITG10 and T2DM + SITG30, effectively reduced (*p* < 0.001) MDA levels to 106.1 ± 5.174 and 71.26 ± 2.101, respectively. Of these two, only the higher dose, T2DM + SITG30, restored MDA levels to values comparable to the control group. Additionally, MDA levels in the SITG30 group (60.95 ± 5.570) were similar to those of the control group.

GSH, a crucial antioxidant, and catalase, which detoxifies ROS, are vital markers of oxidative stress. A depletion in GSH signifies weakened cellular defenses, while impaired catalase function exacerbates oxidative damage in neurons. Both GSH (µmol/g protein) and catalase (ng/g protein) levels were significantly affected by diabetes induction and SITG administration over 30 days (Figure 7B,C). The statistical analysis revealed F(4,25) = 6.384 (*p* < 0.001) for GSH and F(4,25) = 3.937 (*p* < 0.05) for catalase. In diabetes-induced rats, both markers showed a decline (*p* < 0.01) compared to the control, indicating oxidative imbalance. GSH levels dropped to 9.642 ± 0.418 in T2DM rats, while control rats had 18.67 ± 2.195. Catalase levels also declined, with T2DM rats showing 1.352 ± 0.043 compared to 2.232 ± 0.226 in control animals. SITG treatments effectively restored GSH levels, with T2DM + SITG10 at 16.48 ± 0.852 (*p* < 0.05) and T2DM + SITG30 at 17.52 ± 1.361 (*p* < 0.01), but catalase levels remained similar across treatment groups with no significant changes compared to T2DM rats. Notably, SITG30 treatment resulted in GSH and catalase levels comparable to their corresponding control groups.

### 3.7. SITG Treatment Reduced Neuronal Apoptosis in the Brains of T2DM-Induced Rats

**Figure 8:**
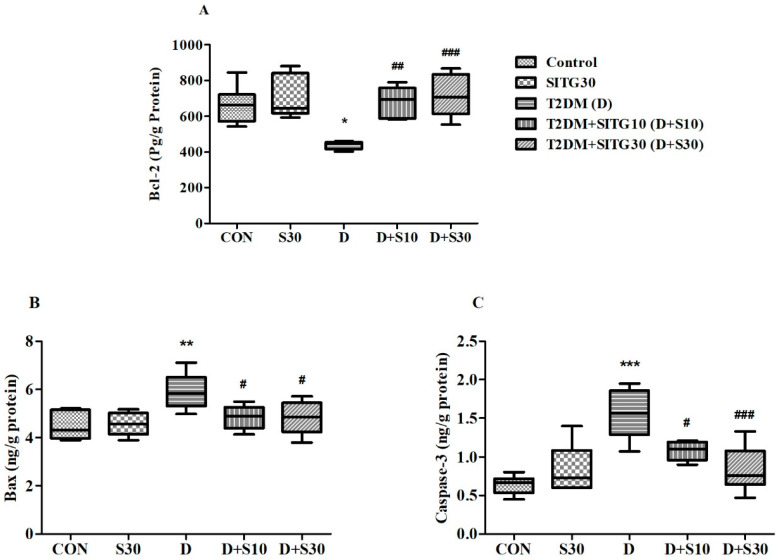
Effect of sitagliptin on apoptotic proteins in the brains of diabetes-induced rats (*n* = 6): (**A**) Bcl-2, (**B**) BAX, and (**C**) Caspace-3. Data are presented as mean ± SEM. * *p* < 0.05, ** *p* < 0.01 and *** *p* < 0.001 vs. Control; # *p* < 0.05, ## *p* < 0.01, and ### *p* < 0.001 vs. T2DM.

In this study, the analysis of three key apoptosis-related proteins was conducted to evaluate the impact of SITG on diabetes-induced neurotoxicity. BCL-2, an anti-apoptotic protein, plays a role in inhibiting cell death and promoting cell survival. Conversely, Bax, a pro-apoptotic protein, facilitates apoptosis by compromising mitochondrial integrity. Caspace-3, another crucial protein, executes the final steps of apoptosis, leading to cell death. These proteins provide insights into the balance between cell survival and death in the context of diabetes and SITG treatment (Figure 8).

In this study, brain Bcl-2 levels (pg/g protein) were significantly influenced (F(4,25) = 8.292, *p* < 0.001) by both the induction of T2DM and the subsequent 30-day treatment with SITG (Figure 8A). The survival of neuronal cells was compromised by diabetes, as reflected in the reduction of Bcl-2 levels (438.7 ± 9.150, *p* < 0.05) in T2DM rats, compared to control rats (662.9 ± 42.67). However, these adverse effects were mitigated following 30 days of SITG administration in diabetic rats, with Bcl-2 levels rising to 634.0 ± 36.06 (*p* < 0.01) in the T2DM + SITG10 group and 716.0 ± 48.31 (*p* < 0.01) in the T2DM + SITG30 group. The SITG group alone showed Bcl-2 levels of 703.1 ± 49.11, slightly higher than control, but without significant difference.

Additionally, both pro-apoptotic protein levels were significantly altered by the treatments in this study: Bax levels (ng/g protein) were affected (F(4,25) = 5.519, *p* < 0.01); Caspace-3 levels (ng/g protein) were impacted (F(4,25) = 11.81, *p* < 0.001) across the different rat groups (Figure 8B,C). Following the induction of diabetes, both proteins were elevated, indicating neuronal apoptosis in the rats’ brains. Bax levels in T2DM rats were 5.912 ± 0.303 (*p* < 0.01) compared to 4.473 ± 0.232 in control rats. Similarly, Caspace-3 levels were significantly increased in T2DM rats (1.557 ± 0.134, *p* < 0.01) compared to controls (0.638 ± 0.049). However, the administration of SITG significantly reduced these pro-apoptotic proteins, demonstrating its neuroprotective effect. Bax levels decreased to 4.845 ± 0.199 (*p* < 0.05) in the T2DM + SITG10 group and 4.823 ± 0.280 (*p* < 0.05) in the T2DM + SITG30 group. Similarly, Caspace-3 levels were reduced to 1.080 ± 0.049 (*p* < 0.05) in the T2DM + SITG10 group and 0.835 ± 0.120 (*p* < 0.001) in the T2DM + SITG30 group. There were no significant changes in Bax and Caspace-3 levels following SITG30 treatment in non-diabetic rats.

### 3.8. Molecular Docking

**Figure 9:**
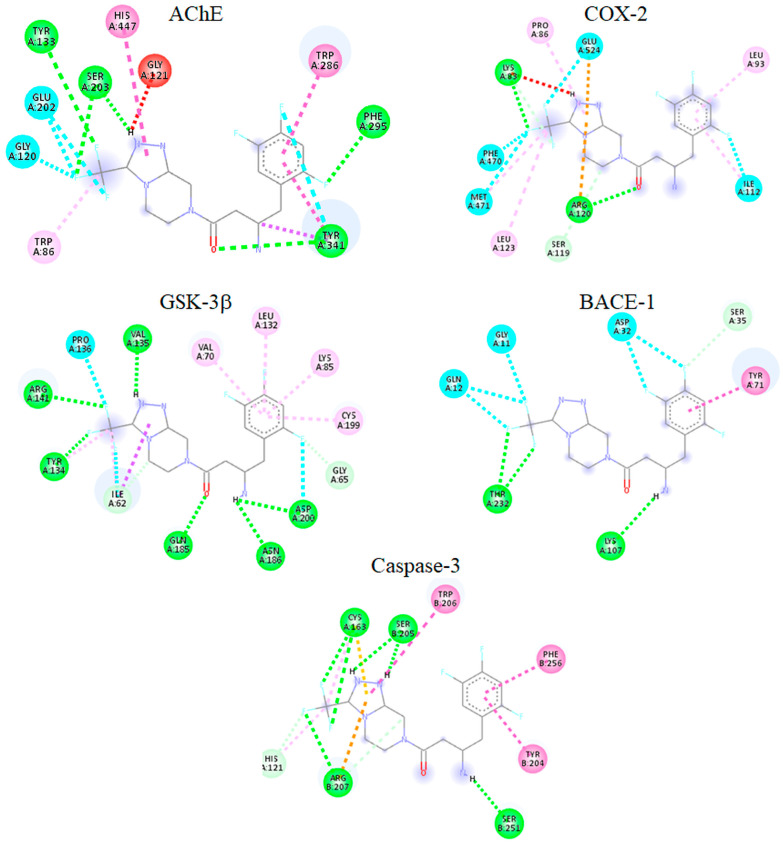
Binding mode of SITG in the active site of *AChE*, *COX-2*, *GSK-3β*, *BACE-1*, and Caspace-3.

Molecular docking was performed to examine the interactions of SITG with *AChE*, *COX-2*, *GSK-3β*, *BACE-1*, and Caspace-3 at the molecular level. SITG showed significant binding scores for *AChE* and *COX-2*, approximately 10.8 and 8.0 kcal/mol, respectively, comparable to those of the co-crystallized ligands. Table 1 summarizes the binding scores of SITG as well as the reference ligands. The co-crystallized ligands demonstrated binding affinities of around 12.1 and 9.1 kcal/mol for *AChE* and *COX-2*, respectively. SITG also exhibited promising affinities for *GSK-3β*, *BACE-1*, and Caspace-3, with binding scores of 9.7, 7.7, and 7.9 kcal/mol, respectively. The binding affinities of the co-crystallized ligands were 9.2, 7.6, and 8.8 kcal/mol for *GSK-3β*, *BACE-1*, and Caspace-3, respectively. Further analysis of the top three low-energy conformations revealed that the most stable conformation of SITG formed interactions with key residues in all complexes. The docked SITG-*AChE* complex showed hydrogen bonds with Tyr133, Ser203, Phe295, and Tyr341, along with halogen interactions involving Gly120, Glu202, and Tyr341. Hydrophobic contacts were observed with Trp86, Trp286, Tyr341, and His447. The SITG-*COX-2* docked complex exhibited hydrogen and electrostatic interactions with Lys83, Arg120, Ser119, and Glu524. Hydrophobic contacts were identified with Pro86, Leu93, Ile112, Leu123, and Met471. Furthermore, halogen bonds were formed with Phe470, Met471, and Glu524. For the docked SITG-*GSK-3β* complex, polar contacts were noted with Ile62,

Gly65, Tyr134, Val135, Arg141, Gln185, Asn186, and Asp200. Hydrophobic contacts were noted with Ile62, Val70, Lys85, Leu132, Tyr134, and Cys199, and halogen bond contacts were noted with Ile62, Tyr134, Pro136, Arg141, and Asp200. The docked complex of SITG with *BACE-1* displayed polar contacts with Ser35, Lys107, and Thr232. Hydrophobic interactions were observed with Tyr71. Further, halogen bonds were noted with residues Gly11, Gln12, Ser35, and Asp32. In the complex of SITG with Caspace-3, polar contacts were noted with His121, Cys163, Ser205, Arg207, and Ser251. Hydrophobic contacts were observed with His121, Cys163, Tyr204, Trp206, and Arg207. Besides, the residues Cys163 and Arg207 were also involved in halogen bonds with the SITG. Figure 9 shows the lowest energy conformation of SITG within the active site of *AChE*, *COX-2*, *GSK-3β*, *BACE-1*, and Caspace-3. Additionally, molecular docking was performed for four commonly used *DPP-4* inhibitors: linagliptin (Lina), saxagliptin (Saxa), alogliptin (Alo), and vildagliptin (Vild). The comparison of binding score showed Lina with similar binding potential as SITG.

## 4. Discussion

In this research, the effects of SITG on various neurodegenerative mechanisms were examined, focusing on key neuronal enzymes associated with dysfunction, including *AChE*, *BACE-1*, *DPP-4*, and *GSK-3β*, as well as neuroinflammation, oxidative stress, and neuronal apoptosis in a diabetic condition. The findings suggest that, in addition to reducing glucose levels through DPP-IV inhibition, SITG may extend its neuroprotective effects in T2DM-induced conditions. This claim is supported by evidence showing the reduced activity of enzymes linked to neuronal dysfunction, along with protection against neuroinflammation, oxidative damage, and apoptosis-related neuronal cell damage. The shared pathological features between T2DM and neurodegeneration, particularly AD, have garnered substantial attention over the past two decades. Further, early evidence reveals that T2DM nearly doubles the risk of developing dementia and AD [23]. Additionally, both conditions become more prevalent with age and have hereditary components. T2DM contributes to AD through several key mechanisms, including oxidative stress, the production of advanced glycation end products (AGEs), chronic inflammation, and autophagic dysfunction. These processes promote the accumulation of Aβ and tau proteins, leading to synaptic dysfunction and microtubule breakdown. Consequently, these factors drive cognitive decline and neurodegeneration, which are hallmarks of AD [4].

Further, GLP-1 receptors are found in several tissues, including the pancreas and brain, where GLP-1 plays a crucial physiological role in regulating blood glucose levels. Previous studies have demonstrated the neuroprotective effects of various GLP-1 analogs in relation to AD [24,25,26]. On the other hand, the *DPP-4* enzyme from several tissues metabolizes the GLP-1 hormone, reducing its effectiveness in controlling glucose levels. By inhibiting the *DPP-4* enzyme, *DPP-4* inhibitors enhance neuronal activity, promoting insulin secretion from pancreatic β-cells and lowering blood glucose levels [18,27,28]. Recent evidence has reported that *DPP-4* inhibitors, including SITG and saxagliptin, reverse AD-like neurodegeneration by enhancing cognitive ability, reducing tau protein and neurofibrillary tangle aggregation, and protecting against Aβ accumulation in an AD mouse model [18]. Therefore, this study further investigates the diabetes-induced AD-related mechanisms of SITG to provide additional supporting evidence.

The experimental T2DM model was induced by a peripheral injection of NICO, followed by STZ administration. STZ selectively destroys insulin-secreting pancreatic β-cells, while prior NICO administration provides partial protection, resulting in T2DM by limiting β-cell damage [19,20]. In pancreatic β-cells, STZ induces DNA damage and activates poly(ADP-ribose) polymerase-1 (PARP-1), leading to the intracellular depletion of NAD+ and ATP and, ultimately, β-cell necrosis. NICO inhibits PARP-1 over activation, preventing excessive NAD+ and ATP depletion, thereby reducing β-cell necrosis and resulting in partial insulin deficiency and hypoinsulinemia [29,30]. In our study, NICO followed by STZ successfully induced T2DM in rats, as indicated by elevated blood glucose and reduced plasma insulin. However, the T2DM + SITG10 and T2DM + SITG30 treatment schedules effectively controlled blood glucose levels and enhanced plasma insulin levels in T2DM rats during the treatment period. Evidence suggests that elevated blood glucose levels, coupled with declining insulin levels, contribute to neurodegeneration and are implicated in AD [31]. Specifically, hyperglycemia and insulin deficiency associated with T2DM can lead to insulin resistance in the brain, reducing neuronal glucose metabolism. This energy disruption accelerates oxidative stress, inflammation, and the accumulation of Aβ and tau proteins, all hallmark features of AD. Additionally, low insulin levels impair the brain’s ability to clear Aβ, exacerbating synaptic dysfunction and cognitive decline. These metabolic disturbances link T2DM with an increased risk for neurodegenerative conditions such as AD [4,31,32].

Our findings on the regulation of blood glucose and plasma insulin levels in SITG-treated T2DM-induced rats underscore the possible neuroprotective effects of SITG against diabetes-induced neuronal degeneration. SITG’s ability to lower blood glucose levels and improve insulin sensitivity is crucial in mitigating the neurodegenerative effects of hyperglycemia, which might accelerate oxidative stress, inflammation, and the formation of AGEs. These factors contribute to neuronal damage, Aβ accumulation, tau protein hyperphosphorylation, and synaptic dysfunction, all key features of AD [4]. In T2DM, insulin resistance in the brain disrupts glucose metabolism and impairs the clearance of Aβ, exacerbating neuronal damage [32]. By reducing blood glucose and enhancing insulin sensitivity, SITG helps restore neuronal energy balance, promote synaptic plasticity, and reduce neurotoxic protein accumulation, potentially slowing neurodegeneration in T2DM-associated AD.

Among targeted enzymes in this study, *AChE* is a key enzyme that plays a specific role in neuronal dysfunction by impacting ACh neurotransmission, which is critical for cognitive functions, especially in the hippocampus, where ACh levels are linked to learning and memory [33]. Located in the neuronal synaptic cleft, *AChE* breaks down ACh, reducing its activity. Elevated *AChE* levels are associated with Aβ plaque accumulation, leading to synaptic dysfunction and neuronal loss [34]. In T2DM, chronic hyperglycemia and insulin resistance may increase *AChE* levels, which impairs ACh signaling, exacerbates insulin resistance in the brain, and heightens cognitive deficit risk. Elevated *AChE* in T2DM also promotes neuroinflammation, increasing susceptibility to AD and other neurodegenerative disorders [35,36]. Our findings indicate that *AChE* levels rose following diabetes induction; however, a 30-day SITG treatment effectively countered this increase, supporting improved cholinergic function in the rat brain. Previously, SITG was shown to restore cyclophosphamide (CYP)-induced *AChE* alterations in the cerebral brain regions of rats [37].

In AD pathology, *BACE-1* is critical in forming Aβ peptides by cleaving the amyloid precursor protein (APP), leading to amyloid plaque buildup, a hallmark of AD that disrupts synaptic function and drives neurodegeneration [38]. In T2DM, chronic hyperglycemia and insulin resistance are linked to increased *BACE-1* levels and activity, as insulin signaling typically regulates *BACE-1*. Under insulin-resistant conditions characteristic of T2D, *BACE-1* activity often rises, increasing Aβ production [39]. This elevated *BACE-1* activity suggests a shared molecular pathway between T2DM and AD, potentially explaining the higher risk of cognitive decline and neuronal dysfunction observed in T2DM patients, as Aβ accumulation is central to both conditions [40,41,42]. Our findings, which demonstrate elevated *BACE-1* levels in T2DM rats, align with this evidence, and the reduction of these elevated *BACE-1* levels in SITG-treated T2DM brains further supports the neuroprotective effects of SITG. Additionally, SITG has demonstrated neuroprotective effects in PTZ-kindling-induced neurotoxicity [28]. Another *DPP-4* inhibitor, linagliptin, has also demonstrated efficacy in reducing Aβ42 accumulation in an AD mouse model [43].

In line with expectations, elevated *DPP-4* levels were observed in the brains of T2DM-induced rats, while both SITG doses effectively reduced these levels in our study. Evidence suggests that increased *DPP-4* levels in T2DM contribute to the progression of AD [11,42]. In T2DM, heightened *DPP-4* promotes insulin resistance and hyperglycemia by degrading GLP-1, potentially impairing cognitive function and elevating AD risk [18]. In AD, elevated *DPP-4* levels are associated with neuroinflammation, oxidative stress, apoptosis, Aβ accumulation, and tau hyperphosphorylation, all of which exacerbate neuronal damage [42]. Studies indicate that *DPP-4* inhibitors can counter these effects by increasing GLP-1 levels, enhancing insulin signaling, and reducing amyloid and tau pathology, underscoring *DPP-4*’s role in both conditions [11,18].

*GSK-3β* is a principal enzyme with diverse cellular functions, and its elevation is linked to the pathogenesis of both T2DM and AD. A comprehensive review explains that impairment in the insulin signaling pathway contributes to AD development by disrupting insulin receptor substrate (IRS) activation. This disruption impedes the activation of the PI3K/AKT pathway, leading to elevated *GSK-3β* levels, which promotes Aβ accumulation, tau protein tangle formation, neuroinflammation, and memory impairment [42]. Moreover, *GSK-3β* over-activity is linked to increased oxidative stress, inflammation, and cell apoptosis within the CNS, all of which contribute to various neurodegenerative conditions. High levels of *GSK-3β* disrupt mitochondrial function and exacerbate neurotoxic processes, leading to a cascade of cellular events that result in neuronal death and dysfunction [44,45]. Additionally, *DPP-4* inhibitors enhance GLP-1 activity, which improves insulin signaling and inhibits *GSK-3β* activity, as insulin activation typically reduces *GSK-3β* levels through the PI3K/Akt pathway [42,44]. Our results demonstrate that T2DM induction leads to elevated *GSK-3β* levels and that inhibition of *DPP-4* by SITG effectively reduces the *GSK-3β* levels induced by T2DM. Furthermore, SITG also countered the increased *GSK-3β* levels in PTZ-kindling-induced rats, facilitating neuroprotective effects [28].

Neuroinflammation is a central feature of neurodegeneration in AD, disrupting neuronal function and leading to cell death. Multiple signaling pathways including NFκB, p38 MAPK, Akt/mTOR, Caspase, nitric oxide, and COX activate immune cells such as astrocytes and microglia, which release pro-inflammatory cytokines such as TNF-α, interleukins, and chemokines, all of which contribute to AD pathology [46]. Additional factors, such as oxidative stress, Aβ accumulation, tau protein hyperphosphorylation, and cholinergic neuron dysfunction, further drive neuroinflammation in AD [47]. Similarly, T2DM induces neuroinflammation through various mechanisms. Elevated peripheral cytokines, including TNF-α and interleukins (IL-1β, IL-2, and IL-6), can cross a compromised blood–brain barrier in T2DM, exacerbating neuroinflammation [4]. Prolonged hyperglycemia in T2DM leads to the formation of AGEs, which accumulate in the brain, binding to receptors on microglia and other cells and activating inflammatory pathways, particularly NF-κB and MAPK) [48,49]. Additionally, impaired insulin signaling within the CNS reduces insulin’s neuroprotective effects, driving microglia into a pro-inflammatory state, leading to sustained cytokine release and increased neuronal vulnerability [50]. Our study measured four inflammation-related markers such as *COX-2*, PGE-2, TNF-α, and NF-κB, all of which were elevated following diabetes induction, reflecting an inflammatory state in neural tissue. SITG treatment effectively reduced these inflammatory markers in the brains of T2DM rats, demonstrating its neuroprotective potential. While GLP-1 receptor expression is generally low in glial cells, it increases in response to brain inflammation, suggesting a role for GLP-1 in controlling inflammation. GLP-1 acts as an anti-inflammatory cytokine, reducing the release of other pro-inflammatory cytokines [51]. The GLP-1 analog liraglutide has been shown to reduce activated microglia and astroglia numbers, as well as pro-inflammatory cytokines IL-1β and TNF-α, in the brains of AD transgenic mice [52,53]. By inhibiting *DPP-4*, SITG enhances GLP-1 activity, impacting inflammatory markers across various experimental models [54]. Notably, SITG has reduced pro-inflammatory markers NF-κB, TNF-α, and IL-6 in diabetic rats with cerebral ischemia/reperfusion injury [55]. In a recent study, SITG demonstrated neuroprotection in a PD rat model, reducing neuroinflammation by lowering levels of TNF-α, IL-6, Iba-1, and GFAP in the brain [56].

Oxidative stress is a critical driver of neuroinflammation, activating pro-inflammatory pathways and causing neuronal damage through excessive ROS. This stress can induce neuronal apoptosis by damaging proteins, lipids, and DNA, ultimately contributing to neurodegeneration and AD [57]. In T2DM, persistent high blood sugar levels enhance ROS production through mechanisms such as glucose autoxidation, protein glycation, and the polyol pathway. Excess glucose within cells can react with proteins and lipids, forming AGEs that further exacerbate oxidative stress [58]. Impaired insulin signaling in T2DM disrupts glucose uptake and leads to mitochondrial dysfunction in neurons. Mitochondria are significant sources of ROS, and their dysfunction results in increased ROS production, which perpetuates a damaging cycle of oxidative stress in the brain and contributes to neurodegeneration [59]. Chronic oxidative stress in T2DM activates microglial cells, which release pro-inflammatory cytokines, creating a neuroinflammatory environment that damages neurons and synapses (essential aspects of AD). Additionally, oxidative stress accelerates the formation of Aβ plaques and tau hyperphosphorylation, further promoting oxidative damage and hastening neurodegeneration [4,60]. GLP-1 has demonstrated antioxidative properties: treatment with GLP-1 receptor agonists significantly reduces oxidative stress markers, including superoxide dismutase (SOD), glutathione reductase, catalase, glutathione peroxidase (GPx), and levels of GSH and LPO, all of which are elevated in response to various stressors [61]. Our results also support the antioxidant effects of SITG in the brains of T2DM rats by reducing MDA levels, a byproduct of LPO, while increasing glutathione (GSH) levels. Additional reports bolster our findings, indicating that SITG reduces oxidative stress by lowering MDA and thiobarbituric acid reactive substance levels while elevating GSH, catalase, SOD, and glutathione peroxidase in neurotoxicity-induced models [62,63].

In details, SITG’s effect on glucose metabolism goes beyond simply lowering blood glucose levels: it plays a critical role in modulating insulin signaling pathways, oxidative stress, and inflammatory responses in the brain, all of which are strongly linked to neurodegeneration. Specifically, SITG enhances insulin sensitivity and promotes insulin signaling pathways, such as the PI3K/Akt pathway, by interfering with *GSK-3β* levels, which are vital for maintaining synaptic plasticity and for neuronal survival. By improving glucose uptake in the brain, SITG helps to restore energy homeostasis and protect neurons from oxidative damage caused by high glucose levels. In parallel, SITG reduces oxidative stress by lowering markers such as MDA and enhancing antioxidant levels such as GSH, which directly alleviates neuronal damage. Additionally, SITG reduces the activation of inflammatory pathways, including NF-κB, *COX-2*, and TNF-α, which are known to contribute to neuroinflammation and accelerate AD pathology. Thus, by improving glucose metabolism, SITG may effectively mitigate the metabolic disturbances that exacerbate neurodegeneration in T2DM-associated AD.

Neuronal apoptosis in neurodegeneration contributes to the progressive loss of neurons and is driven by several interconnected factors, including oxidative stress, mitochondrial dysfunction, excitotoxicity, and inflammation, all of which are prevalent in neurodegenerative diseases [64]. On the other hand, insulin resistance, oxidative stress, and chronic inflammation are associated with T2DM and can drive this apoptotic process, leading to accelerated neuronal loss, cognitive decline, and typical AD pathology [65]. In T2DM, insulin resistance in the brain reduces glucose uptake and utilization, which weakening pathways essential for neuronal survival, synaptic plasticity, and cognitive function. This impairment limits the brain’s neuroprotective mechanisms, making neurons more vulnerable to stress and apoptosis due to reduced energy availability [50,65,66]. In the apoptosis pathway, the Bcl-2 family of proteins plays a critical role in regulating neuronal survival. The anti-apoptotic protein Bcl-2 promotes neuronal survival by stabilizing the mitochondrial membrane and inhibiting the release of cytochrome c. In contrast, the pro-apoptotic protein Bax induces apoptosis by translocating to the mitochondria, leading to membrane permeabilization and the release of cytochrome c. Once released, cytochrome c binds to Apaf-1 to form the apoptosome, which activates Caspace-3, the primary executioner caspase in apoptosis. Activated Caspace-3 cleaves various substrates, executing the apoptotic program and ultimately resulting in cell death [67,68]. Results from the present study, after 30 days of treatment, indicate that SITG promoted anti-apoptosis capability by increasing Bcl-2 protein levels and protected the induction of apoptosis by reducing the Bax and Caspace-3 proteins levels in the brain tissues of T2DM-induced rats. Recently, in traumatic brain injury models, GLP-1 (7–36), an endogenous active form of GLP-1, demonstrated a neuroprotective function against neuroapoptosis by regulating Bcl-2, Bax, and Caspace-3 expression in hippocampal neurons [69]. Moreover, SITG counteracted cerebral neuronal damage induced by PTZ and CYP by controlling the activations of Bax and Caspace-3 proteins levels, supporting the present results [37,62].

Molecular docking is a first-hand, robust approach to predicting the binding affinity and molecular level interaction of small molecules with macromolecules. The predicted binding affinity of SITG against *AChE*, *COX-2*, *GSK-3β*, *BACE-1*, and caspapse-3 was comparable to co-crystallized ligands, indicating the high potential of SITG to bind with the above-mentioned key molecular targets of neuroinflammatory conditions. Further, the binding mode analysis of SITG in the active site of *AChE* demonstrated hydrogen interactions with several key residues including Ser203, His447, and Phe295. Additionally, SITG also displayed a halogen bond with residue Gly120. Further, hydrophobic interactions were noted with Trp286 and Tyr341. It is pertinent to mention that Ser203 and His447 are important residues for the catalysis of ACh, while residue Gly120 is an important member of the oxyanion hole, which, in turn, is important for stabilizing the *AChE*-ACh complex during hydrolysis [21,22]. For the complex SITG with *COX-2*, hydrogen and electrostatic interactions were observed with gatekeeper residues Arg120 and Glu524, respectively, located at the entrance of the hydrophobic tunnel. It is notable that substrate arachidonic acid accessed the oxygenation site through this hydrophobic tunnel. Further, SITG also displayed hydrophobic interactions similar to the crystal structures of NSAIDs with COX [70]. For the docked complex of SITG with *GSK-3β*, polar contacts were observed with Val135, which is important for molecular recognition, while all other residues displaying polar, halogen, and hydrophobic contacts are important for the binding affinity of small molecules [71,72,73]. For the SITG-*BACE-1* complex, SITG displayed hydrophobic and halogen bonds with Tyr71 and Asp32, respectively. Notably, Asp32 plays a key role in the primary inhibition of the enzyme, while Tyr71 is crucial for stabilizing inhibitor binding [74]. For the case of the Caspace-3 docked complex, SITG displayed hydrogen bond contacts with residues His121 and Cys163, both critical for the stabilization of substrate in active sites for hydrolysis [75]. The comparison of binding scores with four commonly used DPP-4 inhibitors indicates that LINA exhibits a binding potential similar to that of SITG across the targets considered in this study.

## 5. Conclusions

This study presents compelling evidence that SITG, a *DPP-4* inhibitor, has significant neuroprotective effects in a rat model of T2DM induced by NICO and STZ. The findings demonstrate that SITG not only effectively manages blood glucose levels but also mitigates key pathological processes associated with neurodegeneration. Specifically, SITG significantly reduces brain enzyme levels linked to AD, including *AChE*, *BACE-1*, *DPP-4*, and *GSK-3β*, while also decreasing inflammatory markers and improving oxidative stress parameters. The above-mentioned results were further supported by the molecular docking results, which indicate significant binding potential and inhibition. These results position SITG as a promising candidate for protecting neuronal integrity in T2DM. Additionally, its ability to enhance anti-apoptotic capabilities underscores its potential in preventing neuronal cell death. Overall, these findings suggest that SITG may serve as a valuable therapeutic intervention not only for T2DM but also for reducing the risk of or delaying the progression of neurodegenerative diseases such as Alzheimer’s. This research underscores the necessity for further clinical investigations to validate SITG’s neuroprotective benefits in human populations and to elucidate the underlying mechanisms by which it exerts its protective effects against neurodegeneration in the context of diabetes. However, this study has certain limitations, notably the absence of molecular dynamics simulations and in vitro evaluations of the target enzymes. Molecular dynamics simulations could have provided a detailed understanding of the binding kinetics and interaction stability, while in vitro assessments would have confirmed SITG activity against the intended biological targets.

## Figures and Tables

**Table 1:** Docking score of SITG and co-crystallized (Co-CL) ligand among different proteins.

Sr. No.	Ligands	*AChE*	*COX-2*	*GSK-3*	*BACE-1*	Caspace-3
1	Co-Cl	12.1	9.1	9.2	7.6	8.8
2	SITG	10.8	8.0	9.7	7.7	7.9
3	Lina	9.0	8.7	8.4	8.6	8.6
4	Saxa	9.6	6.9	6.8	6.5	7.2
5	Alo	8.9	6.5	6.9	6.8	7.3
6	Vilda	9.4	6.8	7.8	6.8	7.3

## Data Availability

The data presented in this study are available from the corresponding authors upon reasonable request. The data are not publicly available due to privacy issues.
